# Traditional herders’ perception of job satisfaction and integration into society: Another obstacle to the survival of pastoralism?

**DOI:** 10.1007/s13280-024-02084-7

**Published:** 2024-10-29

**Authors:** F. Javier Pérez-Barbería, Mark J. Brewer, Iain J. Gordon

**Affiliations:** 1 Biodiversity Research Institute (CSIC, University of Oviedo, Principality of Asturias), University of Oviedo, Campus de Mieres, 33600 Mieres, Spain; 2 Department of Agroforestry Science and Technology and Genetics, IDR, IREC, University of Castilla-La Mancha, 02071 Albacete, Spain; 3 Biomathematics and Statistics Scotland (BioSS), James Hutton Institute, Craigiebuckler, Aberdeen, AB15 8QH Scotland, UK; 4 Fenner School of Environment and Society, The Australian National University, Canberra, Australian Capital Territory Australia

**Keywords:** Livestock ecosystem services, Spain, Sustainability, Traditional husbandry

## Abstract

Pastoralism is gaining in recognition for its provision of a broad range of ecosystem services. However, in Western countries, especially in Europe, it has been in decline for decades and its future is uncertain. Professional satisfaction, social appreciation and community integration are key factors for the sustainability of any activity. Using information from 167 traditional Spanish herders, we assessed their perceived degree of satisfaction with their profession and the reasons behind their perceptions. Herders were extremely satisfied with their profession, but they perceived medium and low levels of appreciation by the rural and urban sectors of society, respectively. Herders had negative feelings about the sustainability of their profession, as they felt more appreciated by society in the past than they do today, half of them did not want their offspring to make a living as herders. Improving communication between herders and society could help to enhance herders’ appreciation of their profession.

**Graphical abstract:**

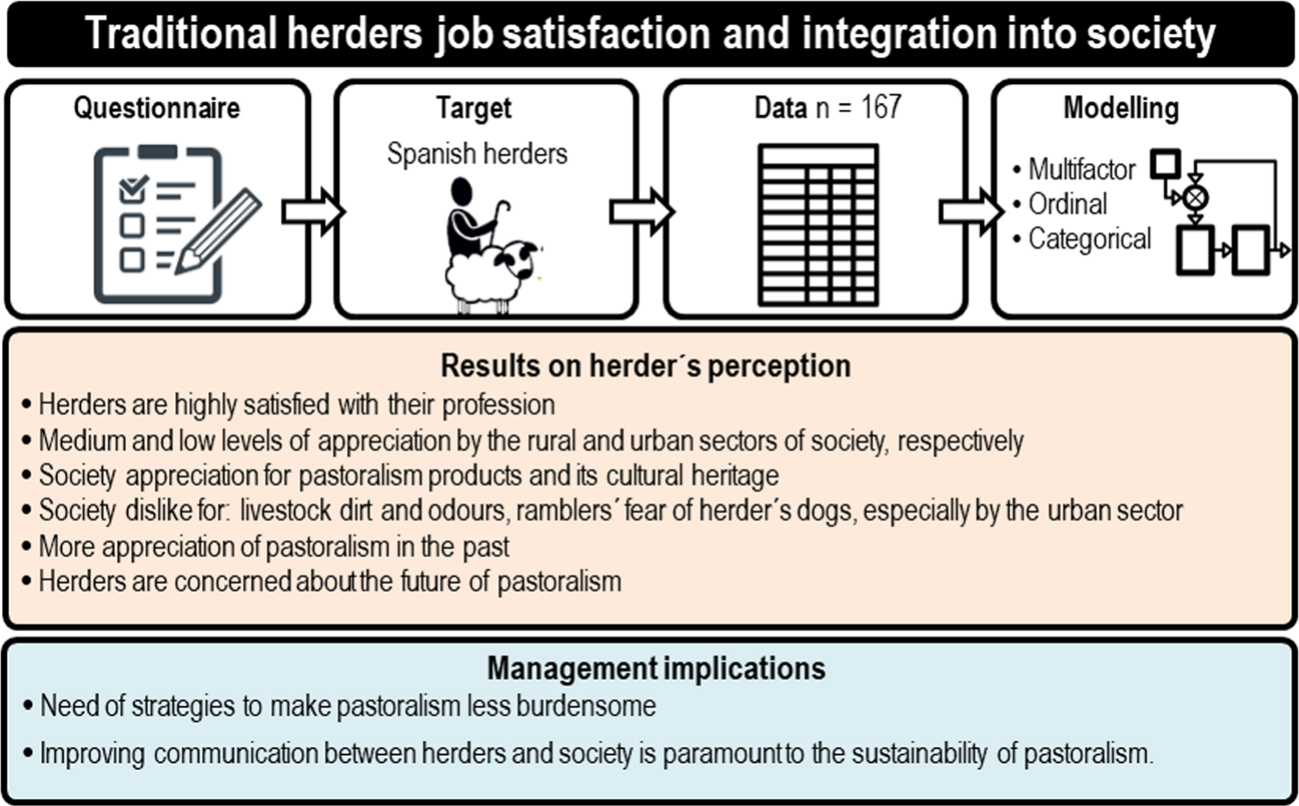

**Supplementary Information:**

The online version contains supplementary material available at 10.1007/s13280-024-02084-7.

## Introduction

Pastoralism contributes to the livelihoods and economies of rural households across the world and is the epitome of healthy rangelands (Zinsstag et al. 2016). However, in the Western countries, pastoralism has been in decline for decades, suffering from the loss of profitability in a global market economy, sedentarization policies, constraints to livestock movement and rural abandonment. Recently, however, pastoralism has been recognized for its social, cultural, economic and ecosystem services provision, including offering women pastoralists the opportunity to hold leading and innovating roles (Oteros-Rozas et al. 2013; Fernandez-Gimenez et al. 2021). It has also having the support of The United Nations, declaring 2026 as the International Year of Rangelands and Pastoralists 1 (FAO 2023).

Small ruminant pastoralism, in southern Europe, is characterized by medium-size herds that graze unfenced marginal grasslands, usually public land and agriculture fallows. These herds are guided by herders, usually in proximity to the farms, to avoid livestock damaging crops or property, causing public liability (e.g., traffic accidents) and over-grazing (Manzano et al. 2023). It should be noted that large distance transhumance movements are not as frequent now as they were in the past (Manzano and Casas 2010; Hadjigeorgiou 2011).

Most pastoralism takes place in developing countries, where millions of pastoralists are neglected by state policies (Dong 2016). This contrasts with Europe where, although pastoralism is on the verge of disappearing, state subsidies, through schemes of the EU Common Agricultural Policy, currently keep pastoralism on life support (Kerven and Behnke 2011). This subsidy policy is justified on the basis that low intensity grazing can support biodiversity, resilience to increasing climate pressures, climate change mitigation, landscape aesthetics, rural economy, cultural heritage and animal welfare (Goddard 2013; Hoffet and Mettler 2017; Gordon and Prins 2019; Wang et al. 2023). Pastoralism’s future is uncertain, despite its benefits to society, because of the low profitability of the products, lack of generational succession, difficulty coexisting with wildlife, particularly predators, hurdles to animal movement and excessive regulations, that have negative effects on the viability of small farms and undermines elder herders’ motivation (Morales-Jerrett et al. 2020; Lecegui et al. 2021; Manzano et al. 2023).

Employees’ satisfaction in their profession is a key element of the performance and sustainability of any business (Chung and Yazdanifard 2014). Key elements contributing to professional satisfaction include their perception of both social integration and the recognition of their activities by society. Identifying these elements in traditional Spanish herders is the main aim of this study and is crucial in developing strategies to improve the satisfaction of the pastoralist guild, and to enhance the future of European pastoralism (Ferguson and Bargh 2004).

We hypothesize that job satisfaction should be high among herders, as vocation is important in pastoralism (Hoffet and Mettler 2017). As we expect that members of rural communities would have a greater level of understanding of animal husbandry, then herders’ perception of appreciation by the rural community should be higher than that held by urban society. We also expected differences in the causes of appreciation or disfavour as perceived by herders between the rural and urban sectors of society, with the urban sector being more appreciative of animal goods versus less tangible features provided by pastoralism, such as cultural heritage or ecosystem services.

## MATERIALS AND METHODS

### Study area

Spain covers an area of approximately 506 000 km^2^, the second-largest country in the European Union, with a population of over 47 million, the fourth-most populous European Union member state.^2^

The total number of ruminant livestock in Spain is 25.7 M (16 M sheep, 2.7 M goats, 7 M cattle) across 181 000 farms (61 000 sheep, 29 000 goats, 91 000 cattle).^3^ It is not known how many of these animals and farms are under a pastoralism system. Pastoralism in Spain has a long tradition, and one notable example is the transhumance guild “Honrado Concejo de la Mesta”, established in 1273 by Alfonso X the Wise. This guild, one of the most significant in Europe during the Middle Ages, granted herders important prerogatives and privileges, such as exemption from military service, freedom of movement and grazing and protection of more than 125 000 km of drover routes, many of which have survived to this day (Ruiz and Ruiz 1986; Manzano and Casas 2010).

Across European countries, Spain leads on a variety of biodiversity indices (e.g., the area of Natura 2000 network, the number of native and endemic species of vascular plants, bryophytes, grasshoppers, birds and mammals) (Sánchez-Fernández et al. 2018). In many protected areas in Spain, pastoralism is present, providing important ecosystem services that can promote and sustain biodiversity (Plieninger et al. 2015; Ruíz and Beaufoy 2015; Olmeda et al. 2018). Spain is also amongst EU Member States that have the greatest agricultural land abandonment in both relative and absolute terms (Perpiña-Castillo et al. 2018).

### Study methods

To understand the complexity of factors that determine the perception of traditional herders of their integration into and value to society, we first selected a non-random focus group that we thought would have a deep understanding of the feelings and opinions of the herders’ collective (Cyr 2019). In this group, we included leaders and technicians of livestock associations and herders with active roles in communication through social media and rural affairs (Focus group in Supplementary information). Using the information derived from this focus group, we constructed a structured questionnaire designed to be conducted on smartphone devices. To ensure that the questions and format were intelligible to traditional herders, we carried out a pilot study, on a small group of herders, and adapted the questions until the users were happy with the format of the questionnaire.

The questionnaire targeted traditional herders that we defined as those whose livestock graze outdoors and rely on natural pasture as the main source of feed. To accomplish this, we asked respondents whether they put their livestock out to pasture on a regular basis. The questionnaire comprised 20 closed and 4 open questions (Questionnaire in Supplementary information). The structure of the questionnaire was: (i) a group of questions to characterize the herder and the farm; (ii) seven questions addressing the degree of appreciation that the herder felt they have from rural and urban society; (iii) sixteen questions (12 closed, 4 open) on the potential causes that made herders  feel appreciated or disfavoured by society; and (iv) one question on the herder’s views of their profession’s generational succession.

To minimize herder conditioning by the previous questions in the questionnaire and “first choice” response bias (i.e., choosing the first option presented), questions and the order of presentation of the response options were randomized across interviewees.

The questionnaire was designed using Microsoft Forms.

#### Questionnaire dissemination

The questionnaire was made available to potential respondents between the beginning of May 2022 and the end of August 2022. Animal farming associations and pastoralism schools across Spain were asked to distribute the questionnaire to their members. Respondents were encouraged to distribute the questionnaire among their fellow herders through a snowballing sampling technique (Goodman 1961). We are aware that the results of the questionnaire could be biased across societal sectors, because (i) this was a self-selected questionnaire, and (ii) it had to be completed online, which would exclude sectors of the pastoralist community with no access to digital technology (Bethlehem 2010). The latter could be less of an issue, as even older generation herders are avid smartphone users, for safety reasons and as a means of entertainment during their work outdoors. Nevertheless, a similar approach has been used in previous studies that have provided valuable information on social trends on nature conservation (Pérez-Barbería and Gordon 2023).

#### Statistical analysis

The questionnaire used two types of variables, ordinal (a rating scale from 1 to 5, from less to most agreement) and categorical. The answers were analysed using linear mixed models controlling for covariates that defined the personal characteristics of the herders and their farms, i.e., gender, age class (≤ 35 yr, between 35 and 51 yr, > 51 yr), being a landowner, coming from herders' family, academic degree (primary, secondary, high), village size, number of livestock.

Because these covariates produced a sparse matrix of data that limited their use in factorial regression type analyses, we summarized their information via the first two dimensions of a multifactorial analysis (MFA) (Husson et al. 2017) applied to the full set of variables that characterized the herder and farm.

To assess the causes driving herders’ perception of societal appreciation of pastoralism, we constructed the following linear mixed model. The response variable was the score of agreement, and the fixed effects were a factor that indicated the question variable and whether herder's perception was for rural or urban society, and the components 1 and 2 of the MFA, and their pertinent interactions. Herder was an additive random effect in the model.

For those variables of categorical nature (questions Q20, Q34–36, Q40–41, in Supplementary Information Questionnaire), we thought of the data as a contingency table and coded a log-linear model. This was carried out by creating a factor that linked the individual questions and the number of potential responses of each (one for each of the number of levels of the categorical response factor) and fitting that in the model as a random effect. The advantage of this approach is that predictions and standard errors should be easily computed. The log-linear model was fitted using the R software package lme4 (Bates et al. 2015), and predictions and associated standards errors using the R package AICcmodavg (Mazerolle 2020).

Because our objective was to identify the main drivers of the response variable, rather than to create predictive models, we used p-values to select the final model in preference to using an information criteria approach (e.g., AIC, BIC) (Murtaugh 2014). The full model was simplified using backward elimination; that is, non-significant fixed-effects terms were removed one at a time following the principle of marginality: the highest-order interactions were tested first, and if they were significant, then the lower order effects were not tested for significance.

MFA was carried out using the R software FactoMineR package (Lê et al. 2008). Statistical analysis was performed using R software (R Core Team 2020), and graphics were constructed using the ggplot2 R package based on The Grammar of Graphics (Wickham 2009).

## Results

### Herders and farms characteristics

We received 167 online completed questionnaires, 17% were from women and 83% from men, and the mean age of herders was 49 years (range = 25–80, Table 1, Supplementary information Results 1).

**Table 1:** Characteristics of herders and farms that participated in the questionnaire

No. of questionnaires	167 (17% women, 83% men)
No. of provinces	35
Herder’s age	mean = 49 years
Raised in herder’s family	70%
Grown up in the local area	66%
No. years as herder	Mean = 25 (range = 5–65)
Level of education	55% basic, 31% medium, 14% high
Herd owner	96%
Sheep herder	54%
Goat herder	8%
Cattle herder	10%
Grazing with donkey	11%
No. sheep	Mean = 714 (range = 20–2500)
No. goats	Mean = 173 (range = 20–1000)
No. cattle	Mean = 109 (range = 5–501)
No. horses	Mean = 10 (range = 1–51)
Village size	Median = 200
Agriculture habitat–natural habitat	70–30%
Production	76% meat, 5% milk, 21% both
Cultivating complementary fodder crops	68%

MFA applied to herder and farm characteristics indicated that 40.5% of the variance was accounted for by the first three components. The scree plot of percentage variance (Supplementary information Fig. S1) shows a relatively slow decline for added components; nevertheless, with only 167 data points, we need to be cautious when including extra variables in the subsequent mixed model analysis. For this reason, we add only the first two MFA components to include the most important variation from the full set of input variables.

**Fig. 1:**
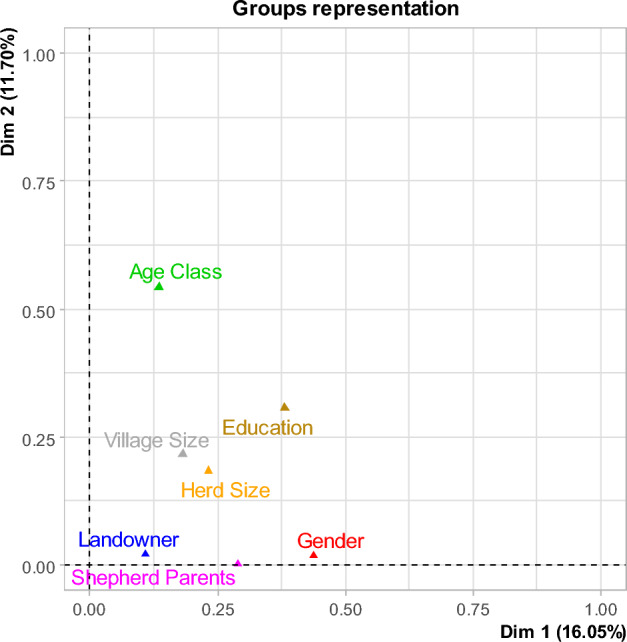
The plot illustrates the correlation between herder and farm characteristics and the first two dimensions of the multifactorial analysis

Age class, gender and level of education best represent the information in the first two dimensions of the MFA (Fig. 1). How the classes of the input variables (gender, age class, level of education, herd size, village size, herding parents, land ownership) are associated with the first two components of the MFA is shown in Supplementary Information Figs. S2–S8 and Results 2.

### Job satisfaction and perception of social integration

**Fig. 2:**
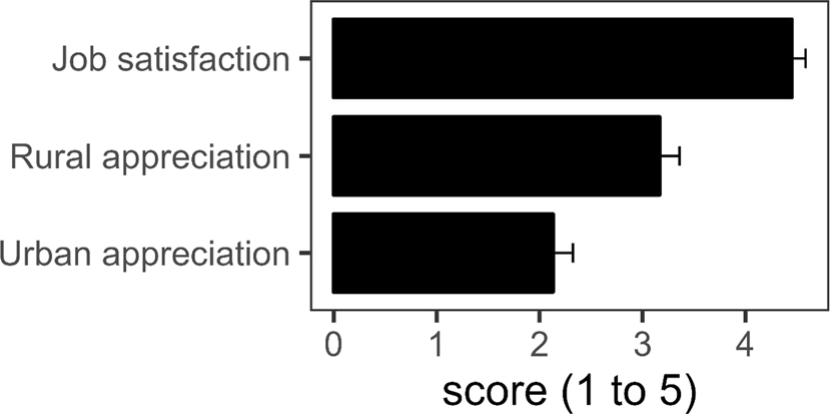
Predicted score and confident interval (95%) of herders’ job satisfaction and the perceived esteem thought to be held by urban and rural sector of society within a rating scale from 1 (low) to 5 (high). From models in Supplementary information Tables S1 and S2

There was a very high level of job satisfaction among herders (estimate of mixed model = 4.5, within a 1–5 rating scale, Fig. 2, Supplementary information Table S1). In the mixed model, neither of the first two components of the MFA were significant, which suggests that the data do not provide any evidence of differences in the herders’ and farm characteristics.

The herders’ perception of their appreciation was on average over one point lower for urban dwellers than for rural people (by rural = 3.2, se = 0.099; by urban = 2.1, se = 0.098); there is very strong evidence for this difference (Supplementary information Table S2, Fig. 2). There is a positive effect for component 2; however, as can be seen by the interaction term, this positive effect is, in effect, cancelled out for the perception of urban dwellers, meaning that the positive effect only holds for the perception from the rural population. There is also very strong evidence for these effects (Table S2).

From Fig. 1, component 2 is most strongly linked to herders’ age and to level of education. High values of component 2 are linked with herders in the youngest age class (Supplementary information Fig. S3); this suggests that there is very strong evidence that the younger herders have a higher perception of appreciation from rural dwellers. Component 2 also distinguishes, to a lesser extent, herders who have an intermediate level of education from those with a high level (Supplementary information Fig. S4). Evidence from this mixed modelling analysis suggests that herders with a high level of education had a higher perception of appreciation from rural dwellers, especially in comparison with herders with an intermediate level of education.

**Fig. 3:**
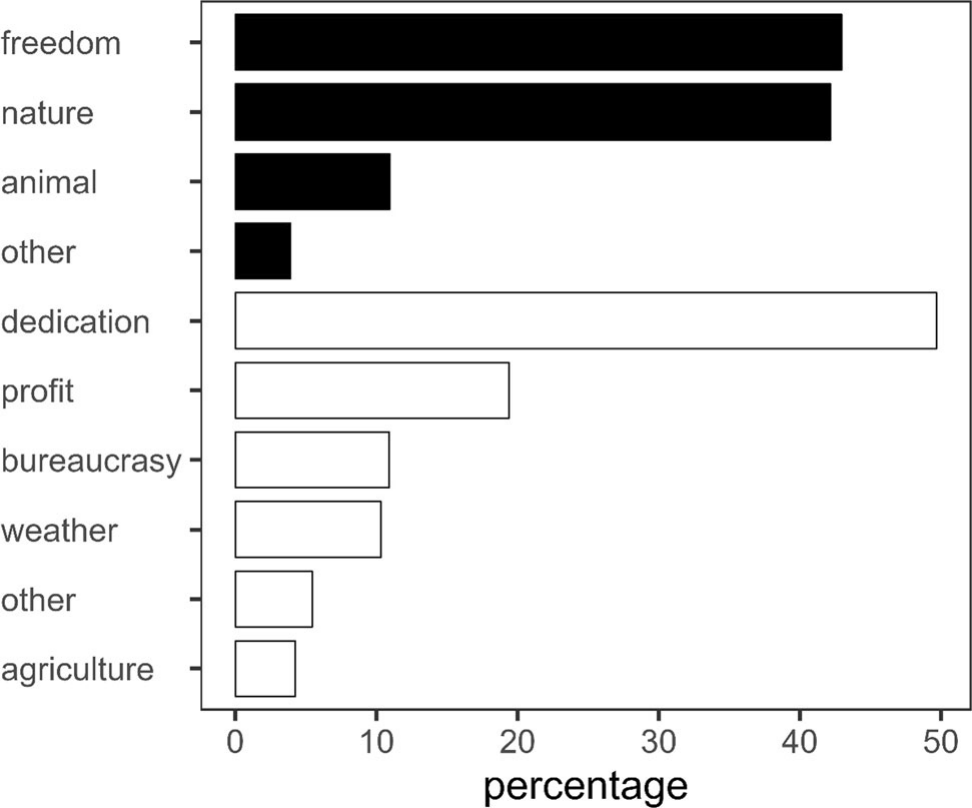
Concept groups of the best (closed bars) and worst (open bars) of being a herder. Percentages are calculated within the total number of answers for “best” and “worst”, separately

The analysis of the open questions helps explain job satisfaction (Fig. 3). Some 43% of the herders thought that the feeling of freedom was the best part of being a herder, using terms like “freedom”, “independence”, “being your own boss”, “developing your own ideas”. Another 42% felt that the best part of the job was working closely with nature, using words like “healthy life”, “nature”, “peace”, and 11% believed that the most satisfying feeling was to work with livestock, using terms like “watching animals growing healthy”, “watching mothers looking after their offspring”, “watching animals graze”. Herders identified hurdles to their profession (Fig. 3) including the enormous dedication required, raised by over 50% of respondents, with terms such as “long working hours”, “no holidays”, “lack of family conciliation”. Some 20% believed that the worst part of the job was the low profitability and price volatility of their goods, while 11% suggested cumbersome bureaucracy and 10% the harsh weather.

### Herders’ perceived causes of appreciation and disfavour of pastoralism by society

**Fig. 4:**
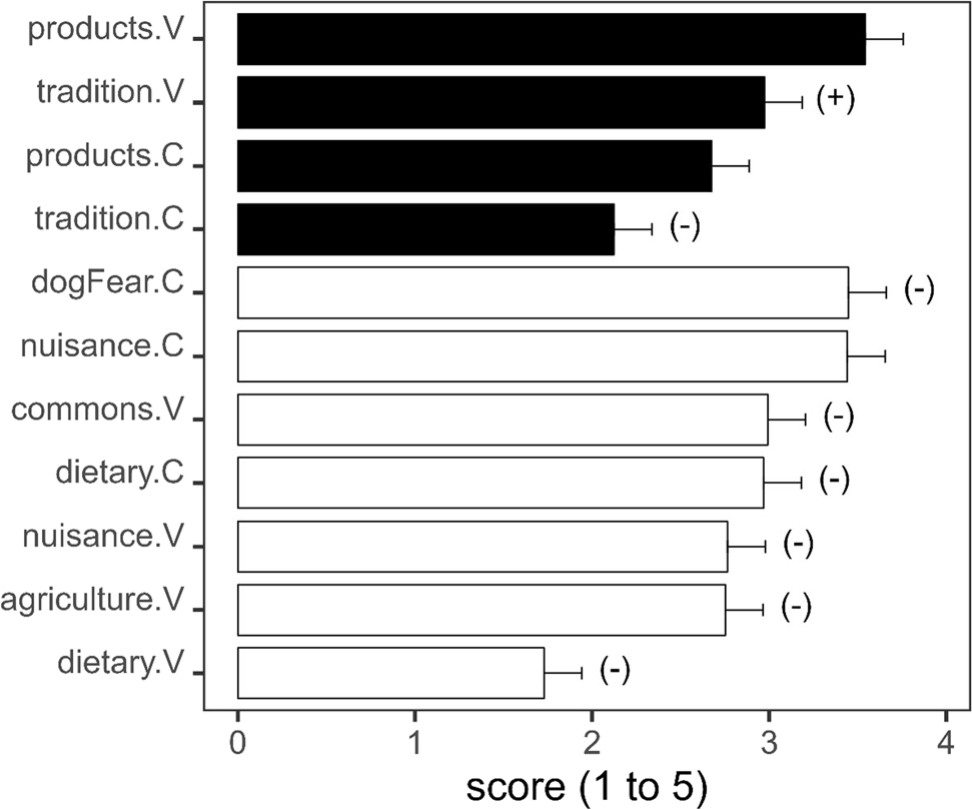
Predicted score and confidence interval (95%) across causes of esteem and disfavour that herders perceive from society (from model in Table 2). Closed bar: cause of esteem; open bar: cause of disfavour. C: as herder believes, it is perceived by urban people; V: as herder believes, it is perceived by rural people. Tradition: cultural heritage; products: livestock products; dietary: social trend towards the reduction of meat in the diet; agriculture: interference with agriculture; nuisance: dirt, odours, noise in villages; dogFear: threat of herder dogs to ramblers; and commons: use of communal pastures. Significant interactions with component 2 of MFA are shown in brackets. Positive interaction means higher scoring by young and high academic degree herders. Negative interaction means higher scoring by mid-aged and middle academic degree herders

Herders’ perception on how society appreciated pastoralism activities and services was complex, as it was not consistent across herder and farm characteristics. This was indicated by the significant interactions of MFA component 2 in the mixed linear model (Table 2, Fig. 4). The most appreciated services were the goods provided by pastoralism and cultural heritage, and these were thought to be more appreciated by the rural than the urban sector (pairwise comparisons rural vs. urban goods *t*-ratio = 5.96, *p* < 0.0001; vs. heritage *t*-ratio = 5.84, *p* < 0.0001, Table 2, Fig. 4). The perception that pastoralism goods are appreciated by society was consistent across herders and farms, as shown by the non-significant effect of MFA components 1 and 2 in the mixed model (Table 2). However, young herders and those with a high academic level felt more strongly that cultural heritage was more appreciated by the rural sector, as shown by the positive effect of the interaction with component 2. The opposite was true for the perceived appreciation of cultural heritage by the urban sector that was scored higher by mid-age and medium academic level herders (Table 2, Fig. 4).

**Table 2:** Predicted scores of herders’ perceived appreciation and disfavour by rural and urban society to different activities of pastoralism. Tradition: cultural heritage; products: pastoralism products; dietary: social trend towards the reduction of meat and milk in people’s diet; agriculture: interference with agriculture; nuisance: dirt, odours, noise in villages; dogs: threat of herder dogs to ramblers; and commons: use of communal pastures. Component 2: second component of MFA. Component 1 was not significant and removed from the model

Predictors	Score estimates	CI (95%)	*p*
Intercept	2.97	2.76 to 3.19	< 0.001
Component 2	0.34	0.15 to 0.52	< 0.001
Products (rural)	0.57	0.28 to 0.85	< 0.001
Nuisance (rural)	0.21	− 0.50 to 0.08	0.151
Agriculture	− 0.22	− 0.51 to 0.06	0.129
Commons	0.02	− 0.27 to 0.30	0.902
Diet (rural)	− 1.25	− 1.53 to − 0.96	< 0.001
Tradition (urban)	− 0.85	− 1.14 to − 0.56	< 0.001
Products (urban)	− 0.30	− 0.59 to − 0.01	0.040
Nuisance (urban)	0.47	0.18 to 0.75	0.001
Dogs	0.47	0.19 to 0.76	0.001
Diet (urban)	− 0.01	− 0.29 to 0.28	0.967
Component 2 × Products (rural)	− 0.10	− 0.35 to 0.15	0.428
Component 2 × Nuisance (rural)	− 0.34	− 0.59 to − 0.09	0.008
Component 2 × Agriculture	− 0.42	− 0.67 to − 0.17	0.001
Component 2 × Commons	− 0.56	− 0.81 to − 0.31	< 0.001
Component 2 × Diet (rural)	− 0.48	− 0.73 to − 0.22	< 0.001
Component 2 × Tradition (urban)	− 0.33	− 0.59 to − 0.08	0.010
Component 2 × Products (urban)	− 0.20	− 0.45 to 0.05	0.121
Component 2 × Nuisance (urban)	− 0.23	− 0.48 to 0.02	0.076
Component 2 × Dogs	− 0.46	− 0.72 to − 0.21	< 0.001
Component 2 × Diet (urban)	− 0.34	− 0.59 to − 0.09	0.008
*σ*^2^	1.8		
Herder	0.19		
Marginal *R*^2^/Conditional *R*^2^	0.137/0.223

High scores of agreement for unfavourable perception were associated with nuisance caused by livestock (dirt, odour and noise), especially by the urban sector (pairwise comparisons urban vs. rural goods *t*-ratio = 5.96, *p* < 0.0001; vs. nuisances *t*-ratio =  − 4.64, *p* = 0.0002, Table 2, Fig. 4) and also the threat of herder dogs to ramblers (score = 3.5, Fig. 4). The perceived nuisance from livestock by urban dwellers was common across herders and farms (non-significant effect of MFA components 1 and 2 in the mixed model, Table 2), but it was mid-aged herders and those with a medium level of academic degree who believed village dwellers complain more about the nuisances of pastoralism, and also that ramblers felt more threatened by herders’ dogs (Table 2, Fig. 4).

One cause of societal disfavour of pastoralism perceived by herders was the trend of changes in dietary habits (i.e. reduction in meat and milk consumption, Fig. 4), especially within the urban sector (disfavour score pairwise comparison: urban = 2.97, rural = 1.73, *t*-ratio =  − 8.507, *p* < 0.0001). This perception was more clearly felt by mid-aged herders and those with a medium academic level, as shown by the significant negative interaction between this factor and MFA component 2 (Table 2, Fig. 4).

Herders believed that interference between pastoralism and agricultural activities and the use of commons were causes of unhappiness with pastoralism within the rural sector. Mid-aged herders and those with a medium level of academic degree showed stronger agreement with these perceptions, as shown by the significant negative interactions between these factors and the MFA component 2 (Table 2, Fig. 4).

Some 69% of herders (se = 6.4) believed that appreciation for pastoralism came mainly from older people versus 29% (se = 4.1) who believed that both old and young people appreciate pastoralism, with only 2% (se = 1.2) believing that younger people appreciate pastoralism the most (Table S3). Also, 68% of herders (se = 6.4) believed they were better appreciated by society in the past than today (5%, se = 1.4), and 27% (se = 4.1) believed that pastoralism was as much appreciated in the past as it is now (Table S4). These two perceptions were consistent across herders and farms (i.e. components 1 and 2 of the MFA were not significant in the models).

When herders were asked about society's preferences for pastoralism versus intensive animal farming products, an equal percentage believed society preferred pastoral products and believed society did not appreciate animal products regardless of the production system (43% and 42%, se = 5.1–5.0, Table S5), 9% (se = 2.3) believed that products from both systems are equally appreciated, and 5% (se = 1.8) that products from intensive farming are preferred by society. These perceptions were consistent across herder and farm characteristics (non-significant effects of MFA components 1 and 2 in the model).

Finally, 48% (se = 4.2%, Table S6) of herders were not keen for their offspring to take up pastoralism as a profession, mainly perceived by mid-aged herders and those with a medium level of education (a significant negative effect of MFA component 2 in the model), while 22% (se = 3.6) would encourage their offspring to become herders, and 30% (se = 5.4) believed they should not interfere in their children’s decision.

## Discussion

### Job satisfaction and perceived social appreciation

Our results show that herders were highly satisfied with their profession, despite this activity requiring full-time dedication, long working hours, loneliness and being outdoors in all weathers (Table 3). Therefore, it would be expected that herders would be ambassadors for their profession within their social circle and that this would permeate through society (Judge et al. 2020). Unfortunately, the continued reduction in the number of traditional herders in Spain (Fernández-Giménez and Ritten 2020) limits the exposure of pastoralism to the public, hindering integration and understanding. A positive association between exposure of an activity to the public and its appreciation by society has been demonstrated in other guilds. For example, US citizens who knew hunters, participated in hunting-related activities or visited fairs or livestock farms had more favourable opinions about hunting than did other people who did not interact with the hunting community (Byrd et al. 2017).

**Table 3:** Summary of results on pastoralists perception of job satisfaction and integration into society, their causes and proposed measures to improve the feeling of being valuable and integration

Herders’ perception	Perceived causes	Implementation measures
Greatly satisfied with their profession	Independence Living in nature Taking care of livestock	Minimize regulatory interference in herders’ activities Respect for the herders’ traditional knowledge on animal welfare and habitat conservation
Low appreciation from the urban sector of society	Nuisances produced by livestock (dirt, odours, noise, insects) Herder’s dogs threat Meat reduction dietary trends	Improve information across society on the ecosystem services of pastoralism to avoid disconnection between rural and urban values and communities Value and respect for tradition-based animal production systems Minimize nuisances produced by livestock Establish channels of communication between herders and society, in which herders make themselves heard Respect for the dietary preferences of everyone and the livelihood of those that produce them
Starting to loss appreciation from the rural sector of society	Interference with agricultural activities Feeling of greediness (i.e. making profit from public land) Nuisances produced by livestock (dirt, odours, noise, insects)	Promote the benefits of the ecosystem services of pastoralism Combat deterioration of the rural social identity Mitigate rural abandonment Value and respect for tradition-based animal production systems
Generationally marginalized	Poorly appreciated by young people	Open pastoralism to school programmes (e.g. school visits to farms)
More appreciated in the past than in the present	Deterioration of the rural social identity	Respect for the rural lifestyle, especially from outsiders (holidaymakers, urbanites, new neighbours) Value pastoralism ecosystem services
Hurdles of job	Full-time dedication Cumbersome bureaucracy Subsidies versus fair price of their products	Implementing mechanisms to alleviate full-time dedication Identify what herders consider “cumbersome bureaucracy” and if possible reduce it Apply policies to avoid unfair competition in products from pastoralism
Uncertain future for the profession	Lack of generational succession	Mitigate rural abandonment Make products profitable See Implementation measures of “Hurdles of the job” above

It is concerning that herders do not have the same feeling of societal appreciation as they have for job satisfaction, especially their perception of appreciation from the urban society. Most herders felt generationally marginalized, i.e., less appreciated by young people than by older people, and herders had a negative feeling of the future of their profession, as they mostly felt that they were more appreciated in the past than they are today (Table 3). Health regulations, aimed at improving people’s health, animal welfare and production, push animal farms away from villages, which might be a cause of herders feeling less integrated than they were in the past, constituting a health-social paradox (Enticott 2008).

There were common causes perceived by herders of societal disfavour of pastoralism, i.e., nuisances produced by their livestock (e.g., dirt, odours, noise, insects), fear of their dogs, interference with agricultural activities, making profit from public resources like communal grassland and receiving economic subsidies. Some of these factors have already been identified as causes of conflict between livestock farmers and people in Western countries. In the Netherlands, for example, annoyance with odours is the main environmental stressor for residents living near animal farms (Hooiveld et al. 2015). In Europe and North America livestock guard dogs wandering and harassing ramblers is a common conflict between herders and society, especially in areas with large wild carnivores where aggressive dogs are required for effective livestock protection (Gehring et al. 2010).

It is remarkable that herders felt that the nuisances caused by livestock came not only from urbanites that visit the village, but also raised by their own neighbours. This is a matter of concern, as it seems to show the deterioration of the social identity of rural communities, a valuable trait for resilience in communities (Ham and Woolcock 2022). It could be claimed that this is not a recent occurrence, but that herders have traditionally conflicted with the community they live in. Witnessing or experiencing social aversion leads to withdrawal, burnout and discouragement of entrepreneurship (Ham and Woolcock 2022), which needs to be addressed to make pastoralism sustainable. In large areas of Spain, where pastoralism took place in agricultural areas, farmers were also owners of livestock, and hired communal herders to look after their livestock belonging. In this scenario, farmers were more permissive of grazing, as they were the direct beneficiaries of the activity, which reduced social conflicts between herders and the community. However, this situation started to change in the 1960s–1970s when herders became owners of the herds (pers. obs.). Almost 70% of our respondents were both herders and farmers, growing their own forage and grain that they use as supplement and bedding. With the use of this practice herders are less dependent on highly priced animal feed and minimize conflicts with farmers as this reduces herders’ dependence on others’ land.

The use of grazing commons is a well-known cause of conflicts in rural communities (Popek 2021). As the number of traditional herders has declined so has the competition for grazing resources, but, paradoxically, our respondents still perceived that a cause of disfavour of pastoralism, by the rural communities, is the use of grazing commons. Here, they raised two main causes of conflict, (i) greediness, herders making profit from public resources, and (ii) farmers interfering in the use of these commons (Supplementary information Focus group). In Spain, herders pay a fee to local farming associations and local administration bodies for the rights of use of commons and stubble and farmers should allow herders enough time to graze stubbles before implementing phytosanitary treatment, ploughing and planting activities, the main cause of conflict between herders and farmers. Farmers and society should be informed that EU subsidizes grazing for the maintenance of grassland and woody pastures which can benefit the conservation of wildlife (Wieren and Bakker 2008; Plieninger et al. 2015; Traba and Pérez-Granados 2022), and that the use of natural roughage resources helps in reducing methane emissions (Pérez-Barbería 2017, 2020; Pérez-Barbería et al. 2020). However, a sector of society puts pressure on pastoralism for its high methane emissions, while disregarding its beneficial ecosystem services, a fair perspective should consider both (Manzano and White 2019).

It should be noted that most of our respondents were sheep herders, and their perceptions might not be representative of cattle pastoralists, whose farming activity is generally easier and less time-demanding (Quaranta et al. 2020).

### Herders’ characteristics and satisfaction

Herders' views of their appreciation by society were complex and were affected by their age and level of academic achievement. In general, middle-aged, middle-educated pastoralists were the most likely to perceive the disfavour of society, while young and highly educated pastoralists felt that urban society appreciated non-tangible services of pastoralism. It is difficult to determine whether this is due to young and highly educated people having a more positive outlook on life, being more proactive in integrating into their community, or simply being less prejudiced (Allport 1958).

It was surprising that there was no gender difference in the job satisfaction or perceived appreciation by society because women tend to be more connected through their social networks than are men (Fernández-Giménez et al. 2022). Women though might be a route through which changed perceptions of pastoralism could be fostered. Pastoralism is a male-dominated profession though women’s contribution has been undermined in developing and western countries despite their active contribution to the running of the farm (Rota et al. 2010; Dong 2016; Fernandez-Gimenez et al. 2021). In our data, 55% of women respondents were actively involved in the administration of the farm, which is a remarkable percentage considering that they were also running the household and other duties. In Europe, there are signs that women are slowly entering the pastoralism profession (Fernandez-Gimenez et al. 2021). Some NGOs are also helping to raise the profile of pastoralist women and their contribution to the production of organic products and habitat conservation (Ganaderas en Red 2023). These are initiatives that help bring pastoralism close to society.

### Intensive and traditional animal farming

Currently, there is a societal belief that traditional animal farming practices pose higher animal welfare standards than those provided by intensive animal husbandry (Boogaard et al. 2011; Alonso et al. 2020), though this is not necessarily true (Stafford and Gregory 2008). Therefore, one would expect herders to believe that urban society would recognize the utilitarian value of pastoralism products from healthy animals that grow in natural environments (Boogaard et al. 2011). This was not the case as herders scored relatively low for their perception of how urban society appreciates pastoralism products in comparison with the perceived appreciation of animal products from the rural community. This emphasizes the need to improve the channels of communication between pastoralism and society to emphasize the value of pastoralism products.

### Herder’s perception of the future of pastoralism

Our respondents were pessimistic about the future of their profession. In Spain, there has been a continuous migration from rural to urban areas since the 1950s. The most recent sharp decline in rural populations has occurred since 2010, mainly in rural areas with less than 1000 inhabitants (Gutiérrez Chacón et al. 2020), which undoubtedly affects pastoralism, since 72% of our respondents live in this type of community. Data from one of the largest Spanish sheep associations (IGP Lechazo de Castilla y León) indicate that between 2021 and 2022 five per cent of their farms closed due to herders reaching retirement age.

Making rural areas an attractive place for young people to live is not easy, as demonstrated by the depopulation rates in these areas. Economic pressures, poor provision of services and deficient public transport in rural areas do not help the situation (Liechti and Biber 2016). Consequently, retaining young people to continue pastoralism-based family businesses or attracting others is unlikely, unless the profitability of the pastoralism products compensates for these hardships. Furthermore, young people from non-pastoralist families, who might want to take up pastoralism, cannot afford the investment required, despite subsides from the government, because they do not own the land and facilities that pastoral households have accumulated across generations.

Regenerative rangeland management has been proposed as a future option for traditional grazing to deliver positive social, economic and environmental impacts (di Virgilio et al. 2019; Serrano-Zulueta et al. 2023). However, it is not clear what is new about this system, other than a rebranding, whether it could provide additional benefits over traditional adaptive grazing techniques (Heady and Child 1994), and how it would improve productivity, while minimizing environmental impacts and labour time.

### Full-time commitment, cumbersome bureaucracy, subsidies and dignification

Full-time dedication, and its associated issues, was a major problem raised by our respondents. Hiring labour could help to cover events, such as sick leave and holidays. This has been implemented in some sheep associations in Castile and León region (Spain), where a herder, working full time for the association, is on standby to cover these events. The initiative is gaining traction among herders in intensive farming systems, but it has limitations in traditional extensive systems where herding requires local knowledge.

Easing cumbersome bureaucracy is a desire of many pastoralists, and this is not a new issue (Schlebecker 1977). Any action plan to reduce bureaucracy should first take into account what herders consider to be “cumbersome bureaucracy” and then analyse how this can be alleviated without compromising the health and safety of people and livestock (Meulen and Freriks 2006). Applying for subsidies adds an extra layer of bureaucracy to pastoralism, for which many herders must rely on service providers, creating in them a feeling of being incapable of running their own business. Several herders raised concerns that living off subsidies was a hurdle for pastoralism, and some suggested that the price of their products should be sufficient to make a living, while others were worried that society might believe that herders make a living from public money. Pastoralism should not survive from subsides, as this is not sustainable in the long term and depends on arbitrary political decisions, but it is difficult to envisage a scenario in which prices of non-subsided eco-friendly pastoralism products can compete against products produced by intensive animal farming (Scown et al. 2020).

Our results clearly indicate that there is a dissatisfaction of herders because of their perceived appreciation of their activity by society, and whether this is a real reflection of what society feels about pastoralism is a different matter. Lack of communication and societal segregation are common causes of prejudice (Allport 1958). Communication between society and herders could be improved so that herders have a positive perception of society’s sentiment towards pastoralism, but this might not be enough for herders to feel valued (Table 3). The actual trend of pastoralism as a tool for the conservation of cultural landscapes and biodiversity is increasing, as such making herders participants in decision making programmes of habitat conservation, as a recognition and respect of traditional knowledge on management practices, could help to improve their integration into society (Molnár et al. 2023).

## Conclusions

Although traditional herders were very satisfied with their profession, they raised issues concerning a dissociation between themselves and the urban and rural sectors of society and signs of deterioration of their social identity within rural communities, where herders feel neighbours disapprove of some externalities of the profession. The results of this study indicate that there is a need to (1) improve the esteem in which herders are held by society, and (2) assess the perception that society has of pastoralism, to know how both sectors of society perceive each other. The former could be achieved through better communication between herders and society. Thereby, society would be informed of the beneficial ecosystem services provided by pastoralism, to better understand and improve tolerance towards pastoralism. If society idealizes rural life without respecting its activities, traditions and way of life, we will end up turning our villages into theme parks with no room for traditional pastoralism.

## Supplementary Information

Below is the link to the electronic supplementary material.

Supplementary file1 (PDF 605 KB)

## Footnotes

1 https://iyrp.info/.

2 https://ec.europa.eu/eurostat accessed 14/03/2023.

3 https://www.ine.es accessed 24/03/2023
