# Supplementary material for: Traditional herders’ perception of job satisfaction and integration into society: Another obstacle to the survival of pastoralism?
Source: Ambio. 2024 Oct 29;54(2):325–37. doi: 10.1007/s13280-024-02084-7 (PMC11662114; doi:10.1007/s13280-024-02084-7)
Supplement: Supplementary file 1 — Supplementary file1 (PDF 605 KB) [file 13280_2024_2084_MOESM1_ESM.pdf]

Ambio

Supplementary Information

This supplementary information has not been peer reviewed.

Title: **Traditional herders´ perception of job satisfaction and integration into society: another obstacle to the survival of pastoralism?**

Questionnaire

Presentation

We are the Biodiversity Research Institute from the Spanish National Research Council, and we would like to know what is your perception about the appreciation that society has towards you, the traditional herders, which can help the sustainability of the profession. We would be very grateful if you answer this questionnaire.

Your consent to participate

Q1. In order to start the survey, please read the following paragraph and click whether you agree or disagree.

- I am participating in this research voluntarily and I know that I have the right to withdraw from this research at any time without explanation.
- I have been notified that, if the information from this research is used in a scientific publication, my personal data will not be shown.
- I have been informed that no one will know what I answered.

Yes, I accept the conditions of participation and wish to continue with the questionnaire.
No, I do not accept the conditions of participation and do not wish to continue with the questionnaire.

About you and your livestock farm

Q2. Do you regularly graze your livetock?
Yes / No, my livetock is almost always in stalls.

Q3. Do you like being a herder?
Please rate between 1 (I like being a herder very little), 2, 3, 4, 5 (I like being a herder very much).

Q4. What livestock do you have (you can check more than one box)?
Sheep / Goats / Cows / Horses

Q5. Are you the owner of the livestock?
Yes / No

Q6. Age (in years)

Q7. Sex
Female / Male

Q8. Name of the village where you have your farm

Q9. Do you come from a herder family?
Yes / No

Q10. How many inhabitants live in the village throughout the year?

Q11. In which province do you live?

Q12. Is your farm in an area that is predominantly agricultural?
Yes / No

Q13. What education do you have?
Basic / Middle school / High school

Q14. How many sheep do you have?

Q15. How many goats do you have?

Q16. How many cows do you have?

Q17. How many horses do you own?

Q18. What are you a producer of, meat, milk or meat and milk?
Meat / Milk / Meat and milk

Q19. Do you cultivate your own land?
Yes / No

About what you think

Q20. Do you feel appreciated in the village?
Yes, I am appreciated as a neighbour and as a herder /
I am appreciated only as a neighbour /
I am appreciated only as a herder /
I am neither appreciated as a neighbour nor as a herder.

Q21. Do people in town appreciate herder?
Rate between 1 (very little), 2, 3, 4, 5 (very much)

Q22. Do people in the city appreciate herders for their image and tradition?
Please rate between 1 (very little), 2, 3, 4, 5 (very much)

Q23. People in the city appreciate herders for the products they produce.
Scores between 1 (very little), 2, 3, 4, 5 (very much)

Q24. City people do not appreciate herders because when they go on vacation to the villages livestock bother them and litter the streets.
Rate between 1 (I do not agree), 2, 3, 4, 5 (I completely agree).

Q25. City people do not appreciate herders because when they are riding bicycles or walk along the roads herders' dogs threaten them.
Rate between 1 (I do not agree), 2, 3, 4, 5 (I completely agree).

Q26. City people do not appreciate herders because city people do not

agree with animals being used for human consumption.
Rate between 1 (I do not agree), 2, 3, 4, 5 (I completely agree).

Q27. Do village people appreciate herders?
Rate between 1 (I do not agree), 2, 3, 4, 5 (I completely agree)

Q28. Village people appreciate herders for their image and tradition.
Please rate between 1 (I do not agree), 2, 3, 4, 5 (I completely agree).

Q29. Village people appreciate herders for the products they produce.
Rate between 1 (I do not agree), 2, 3, 4, 5 (I completely agree)

Q30. Village people do not appreciate herders because livestock disturb people and litter the streets
Rate between 1 (I do not agree), 2, 3, 4, 5 (I completely agree).

Q31. Village people do not appreciate herders because herders interferes with agricultural work
Rate between 1 (I do not agree), 2, 3, 4, 5 (I completely agree).

Q32. Village people do not appreciate herders because they think that herders live at the expense of communal pastures or of resources from farmers' land (fallow land, stubble fields)
Rate between 1 (I do not agree), 2, 3, 4, 5 (I completely agree).

Q33. Village people do not appreciate herders because village people do not agree with animals being used for human consumption.
Rate between 1 (I do not agree), 2, 3, 4, 5 (I completely agree).

Q34. When were herders more appreciated?
Before / Now / Same

Q35. Who appreciates herders the most?
Old people / Young people / Same

Q36. Do you have more problems with villagers or with vacationers?
I have more problems with villagers /
I have more problems with vacationers /
I have the same problems with both /

Q37. What do villagers complain most about the herders?

Q38. What do city people complain most about herders?

Q39. What makes your life as a herder most difficult?

Q40. Who does society appreciate the most?
Pastoralism / Intensive animal farming / Both / Neither

Q41. Would you like your children to be a herder?

Yes / No / It is not at to me

Q42. Why would or would not you like your children to be herders?

Q43. What is the best thing about being a herder?

Q44. What is the worst thing about being a herder?

Survey to be answered by herder´s partner

Here questions Q1, Q3, Q5-7, Q9, Q13, Q20-44.

Q45. In which of these tasks do you collaborate (you can mark more than one task)?
I collaborate in indoors work of the farm
I collaborate in guiding the livestock when grazing
I collaborate in the administrative management of the farm
I collaborate in housework
I have my own work away from the farm

Focus group (discussion protocol)

Objective:

The objective of this focus group discussion is to explore the perceptions, experiences, and factors related to job satisfaction and integration into society among traditional herders. The insights gathered will contribute to a better understanding of the challenges they face and potential strategies to improve their overall well-being.

Participants:

We selected a 4 non-random group of traditional herders that we thought would have a deep understanding of the feelings and opinions of the herders´ collective.

Discussion Guide:

1. Introduction (6 minutes):

- Welcome and introductions.

- Briefly explain the purpose of the discussion and the importance of their perspectives.

2. Data Privacy (1 minute):

- Ensure the confidentiality and anonymity of participants in any subsequent reports or publications.

3. Icebreaker (5 minutes):

- An icebreaker question to help participants feel comfortable and build rapport.

5. Main Discussion (30 minutes):

- Use of open-ended questions to encourage participants to share their experiences and perceptions and encourage participants to respond to each other's comments.

  - Job satisfaction: What aspects of herding do they find satisfying or dissatisfying?

  - Integration into society: How do they perceive their role in the community?

  - Challenges and barriers: What obstacles do they face in their daily lives?

  - Coping strategies: How do they manage stress and difficulties?

  - Suggestions for improvement: What changes would they like to see to enhance their job satisfaction and integration into society?

6. Summary (5 minutes):

- Summarize the key points discussed and ask participants if there is anything they would like to clarify or add.

7. Closure (2 minutes):

- Thank participants for their valuable insights.

- Provide information about the next steps in the research process.

Facilitation:

- Encourage all participants to speak and listen actively.
- Keep the discussion on track and within the time limits for each section.

Focus Group. Results.

Job satisfaction: What aspects of herding do they find satisfying?

Those aspects related to the actual activity of the job, mainly, independence, living in the backcountry, breeding livestock and animal welfare.

Job satisfaction: What aspects of herding do they find dissatisfying?

Issues related to long working hours; cumbersome bureaucracy; lack of support from the administration.

Integration into society: How do they perceive their role in the community?

Feeling of being a minority in society; no leverage to influence market prices.

Challenges and barriers: What obstacles do they face in their daily lives?

Problems in interactions with holidaymakers and newcomers; problems with agriculture farmers; use of communal pastures; lack of generational replacement.

Coping strategies: How do they manage stress and difficulties?

Highly variable depending on personal circumstances.

Suggestions for improvement: What changes would they like to see to enhance their job satisfaction and integration into society?

Better prices of their products; less regulations and bureaucracy; improving societal understanding of pastoralism and rural affairs; greater appreciation of their profession; reduce farming – wildlife conflicts.

Results 1

Questionnaires were answered from 35 out of the 50 Spanish provinces, which covers all Spanish inland regions and the insular Canary archipelago. The largest numbers of questionnaires were from the regions of Castile and Leon (38%), Aragon (15%) and Andalusia (10%).

Due to the limited sample size and the variety of herder and farm characteristics, we refrained from including any regional factors in the analysis. Twenty-five percent of the questionnaires came from populations of fewer than 51 inhabitants. Seventy percent of herders were raised in herders´ families and were locals (66%), 55% had completed studies at level 2 and 45% had levels of education > 2 (International Standard Classification of Education). Most questionnaires came from sheep herders and a smaller number from goat and cattle herders or those with mixed species herds (Table 1), mainly dedicated to meat production. Sixty-eight respondents were also farmers that grow their own forage to supplement the feeding of their livestock. There were forty-two herders that were women that filled in the questionnaire, most of them participated in the running of the farm with more than one task, 62% shared household and farm duties, 55% were involved in the administration of the farm, 31% guided the herd during grazing, 24% had their own job, and 7% were fully dedicated to household activities.

Results 2

The data shows that there was an association between education level attained and gender; although only 17% (29) of the 167 people in the sample were women, 35% (8) of the 23 for the overall sample with high level of education were women. Correspondingly, a greater proportion of men attained only a basic level of education. Men were 3 times more likely than women to have parents who were herders (no herding parents = 35 men, 15 women; herding parents = 103 men, 14 women). Herders from herding families were much more likely to have only a basic level of education (basic = 73, intermediate = 29, high = 15) than herders from non-herding families (basic = 19, intermediate = 23, high = 8). In fact, this effect was almost entirely caused by differences in men; the relatively small numbers of women in the sample were equally divided between being from a herding or non-herding family for all three levels of education.

Tables

Table S1. Coefficients of a linear mixed model of herders´ profesional satisfaction. The response variable is a rating scale from 1 “low satisfaction” to 5 “high satisfaction”. Component 1 and 2 are covariates that represent the first two dimensions of a multifactorial analysis that summarises characteristics of herder and farm. CI: confident interval.

Predictors	Score estimates	CI (95%)	p
(Intercept)	4.45	4.31 – 4.59	**<0.001**
Component 1	0.06	-0.04 – 0.16	0.234
Component 2	0.04	-0.08 – 0.15	0.537
Random Effects			
σ^2	0.14		
Herder	0.64		
Marginal R^2 / Conditional R^2	0.011 / 0.826		

Table S2. Coefficients of a linear mixed model of herders´ perceived appreciation by people from “rural” and “urban” sectors of society. The response variable is a rating scale from 1 “low appreciation” to 5 “high appreciation”. Reference level is “rural”. CI: confident interval. Component 1 and its interaction with urban/rural factor were not significant and removed from the model.

Predictors	Score estimates	CI (95%)	p
(Intercept)	3.17	2.97 – 3.36	**<0.001**
Urban	-1.04	-1.23 – -0.84	**<0.001**
Component 2	0.33	0.16 – 0.50	**<0.001**
Urban × Component 2	-0.36	-0.54 – -0.19	**<0.001**
Random Effects			
σ^2	0.83		
Herder	0.72		
Marginal R^2 / Conditional R^2	0.180 / 0.561		

Table S3. Coefficients of a categorical linear mixed model on herder´s perceived appreciation from different age sectors of society. Herders were asked to choose if they perceived more appreciation from old people “old”, young people “young” or similar between old and young people “same”. Reference level is old people.

Predictors	Incidence Rate Ratios	CI	p
(Intercept)	0.69	0.57 – 0.83	**<0.001**
Same	0.42	0.30 – 0.58	**<0.001**
Young	0.03	0.01 – 0.09	**<0.001**
Random Effects			
σ^2	1.91		
Marginal R^2 / Conditional R^2	0.515 / NA		

Table S4. Coefficients of a categorical linear mixed model on herder´s perceived appreciation from the society over time. Herders were asked to choose if they perceived they were more appreciated in the past “past”, in the present “present” or similarly over time “same”. Reference level is “past”.

Predictors	Incidence Rate Ratios	CI	p
(Intercept)	0.68	0.56 – 0.81	**<0.001**
Present	0.07	0.03 – 0.15	**<0.001**
Same	0.41	0.29 – 0.57	**<0.001**
Random Effects			
σ^2	1.74		
Marginal R^2 / Conditional R^2	0.410 / NA		

Table S5. Coefficients of a categorical linear mixed model on herder´s perceived appreciation from society for animal farming products. Herders were asked to choose if they perceived more appreciation from society for products from intensive animal farming “intensive”, “pastoralism”, “both” or “none”. Reference level is “both”..

Predictors	Incidence Rate Ratios	CI	p
(Intercept)	0.09	0.05 – 0.15	**<0.001**
Intensive	0.60	0.26 – 1.37	0.226
None	4.73	2.71 – 8.26	**<0.001**
Pastoralism	4.80	2.75 – 8.37	**<0.001**
Random Effects			
σ^2	1.90		
Observations	668		
Marginal R^2 / Conditional R^2	0.311 / NA		

Table S6. Coefficients of a categorical linear mixed model. Herders were asked to choose if they preferred their offspring to make a living as herder "Yes", not making a living as herder "No", or "Indifferent". Reference level is "Indifferent".

Predictors	Incidence Rate Ratios	CI	p
(Intercept)	0.29	0.22 – 0.39	**<0.001**
No	1.61	1.12 – 2.31	**0.011**
Yes	0.72	0.46 – 1.12	0.146
Indifferent × Component2	1.24	0.98 – 1.57	0.075
No × Component2	0.78	0.64 – 0.96	**0.018**
Yes × Component2	1.23	0.93 – 1.62	0.146
Random Effects			
σ^2	1.42		
Marginal R^2 / Conditional R^2	0.108 / NA		

Figures

Figure S1

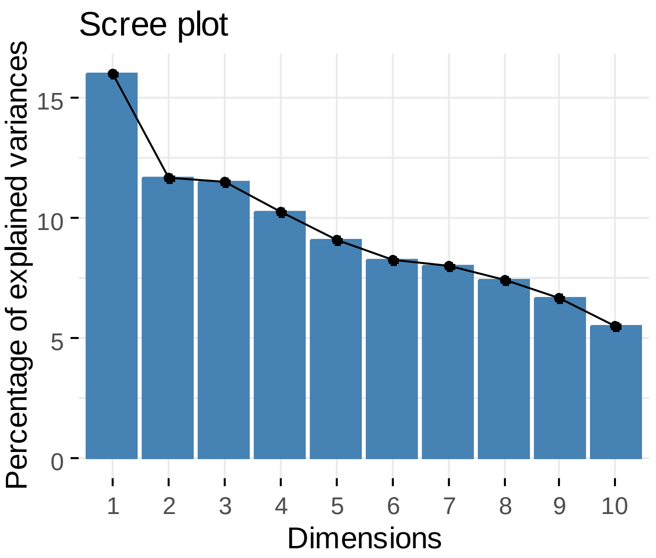

Figure S1. Scree plot of percentage variance for added components of the MFA analysis on herder and farm characteristics.

Figure S2

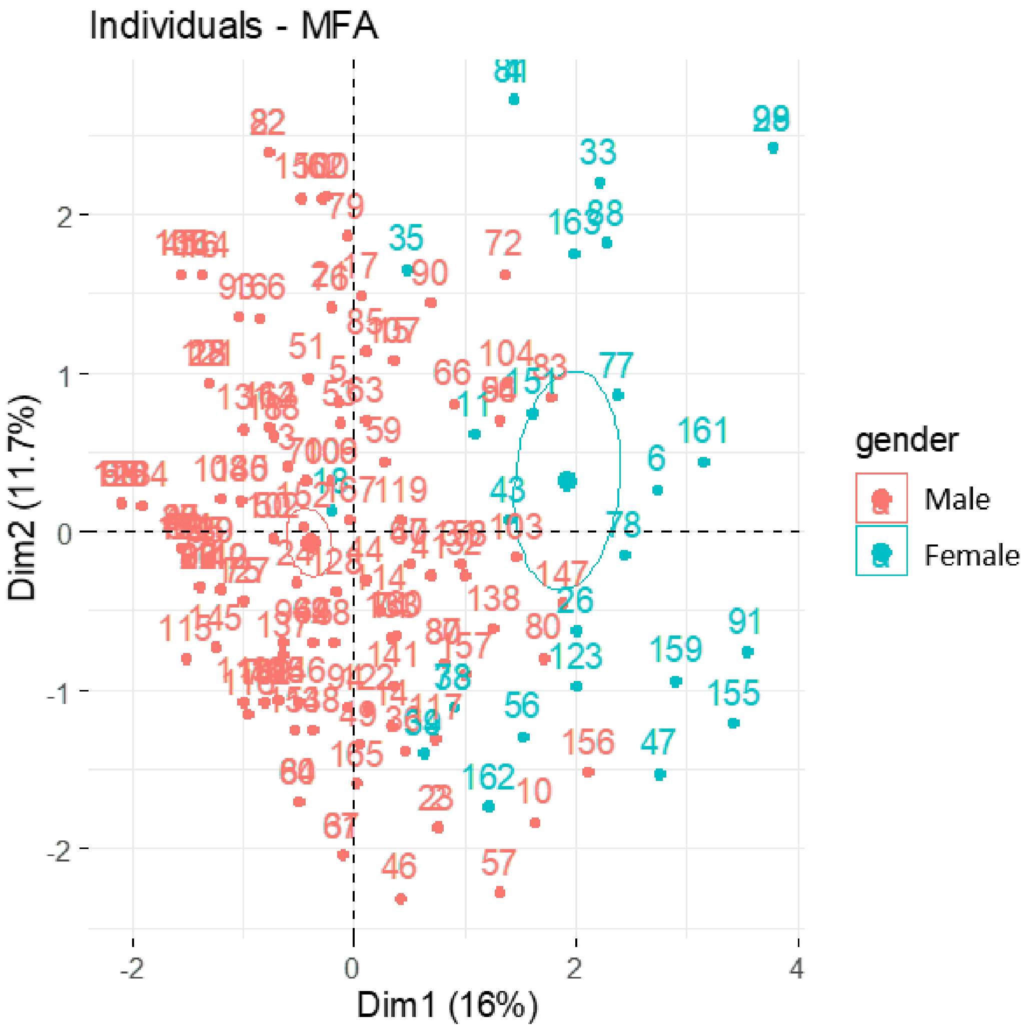

Figure S2. The plot shows that the first component of the multifactorial analysis is strongly associated with gender.

Figure S3

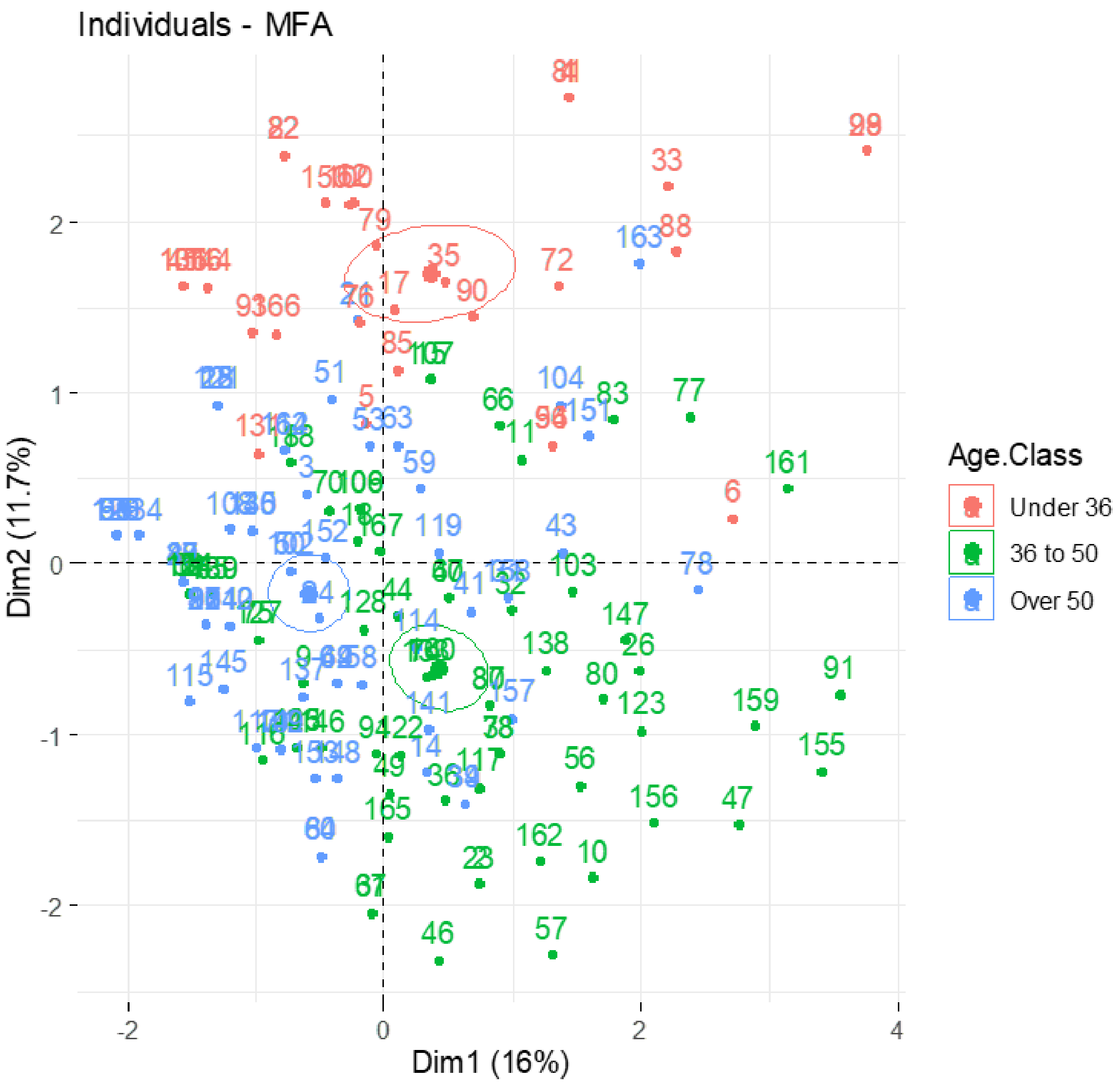

Figure S3. The youngest age class is associated with high values of component 2 of the multifactorial analysis, while to a lesser extent, component 1 separates out the two other age classes, with lower values associated more with the oldest age group.

Figure S4

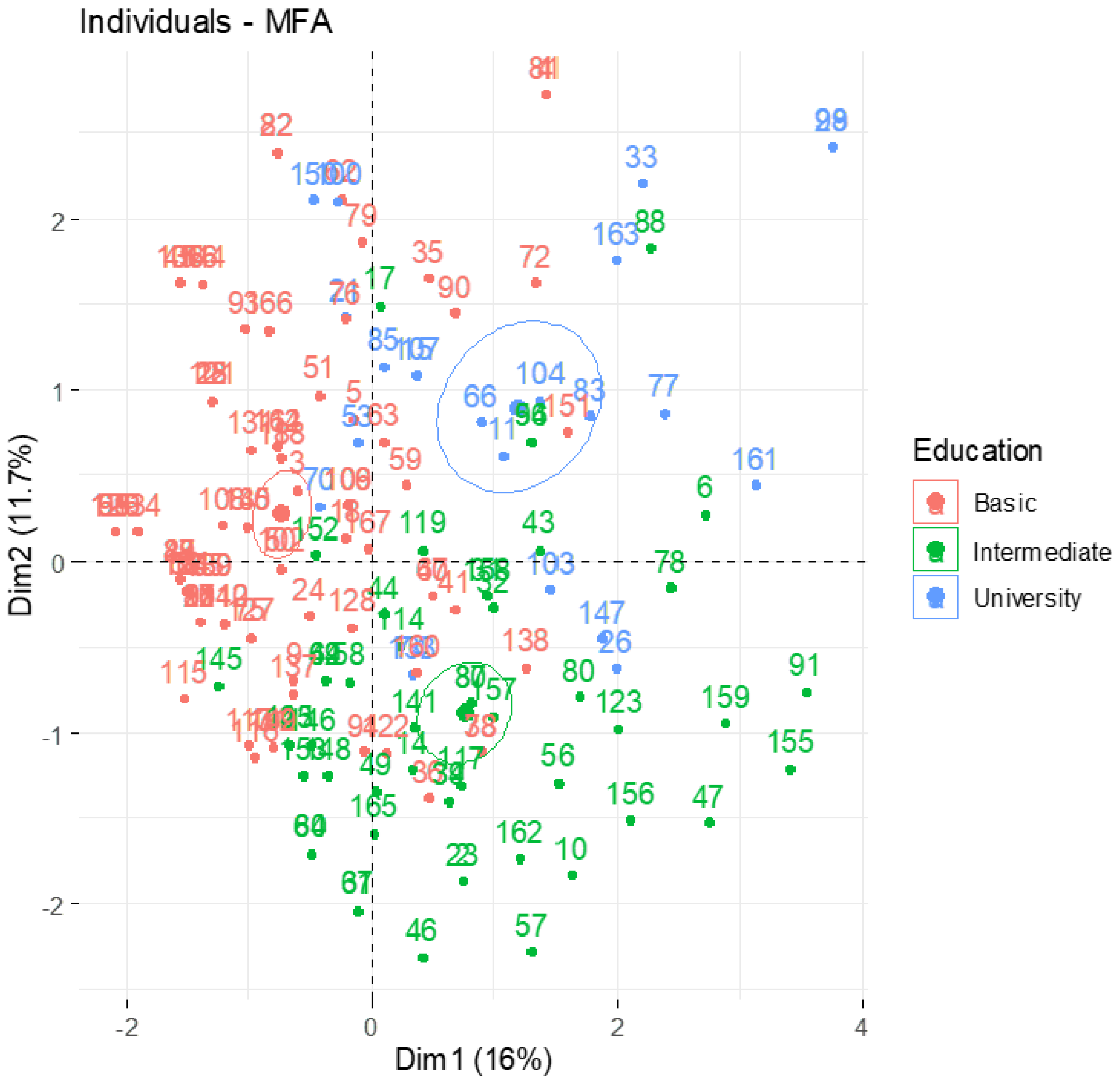

Figure S4. We can see that having a university degree is linked to high values of both components 1 and 2 of the multifactorial analysis, while generally people with a basic level of education tend to have low values of component 1, and people with the intermediate level of education tend to have lower values of component 2.

Figure S5

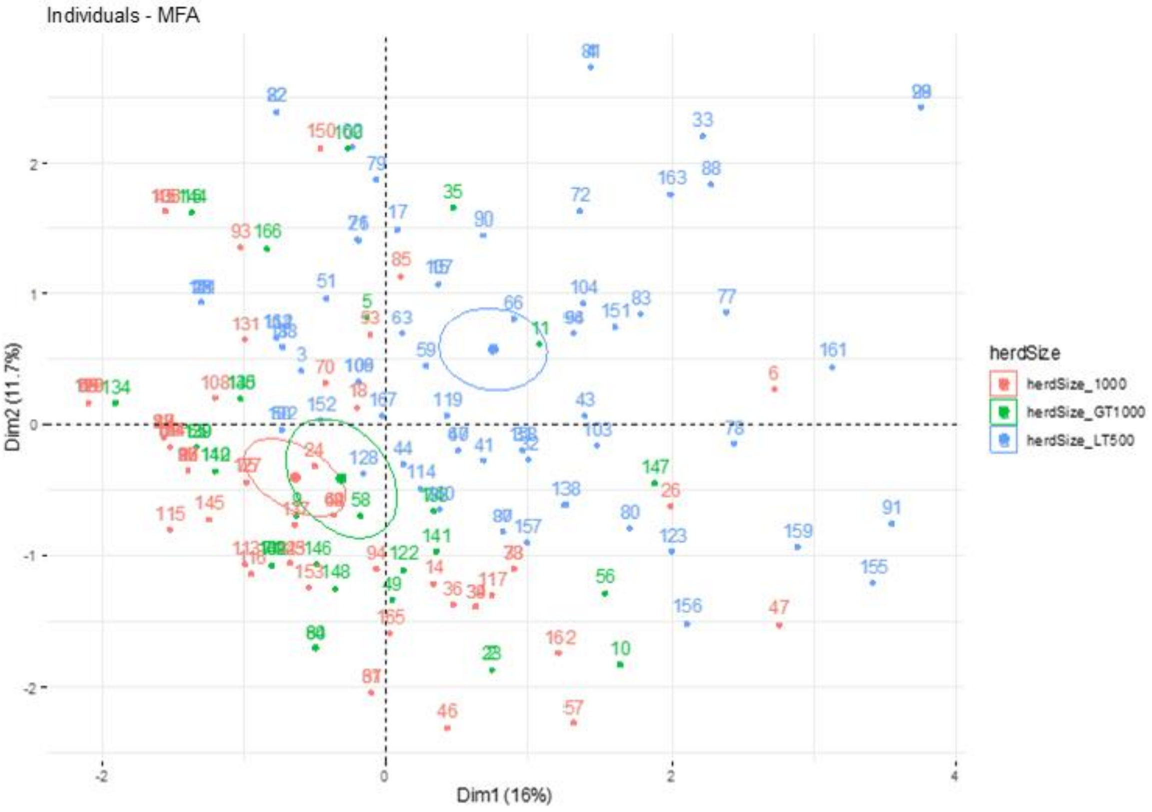

Figure S5. The plot shows that the larger herd sizes are linked with higher values of both components of the multifactorial analysis (LT500 = less than 500 animals; 1000 = between 500 and 1000 animals; GT1000 = more than 1000 animals).

Figure S6

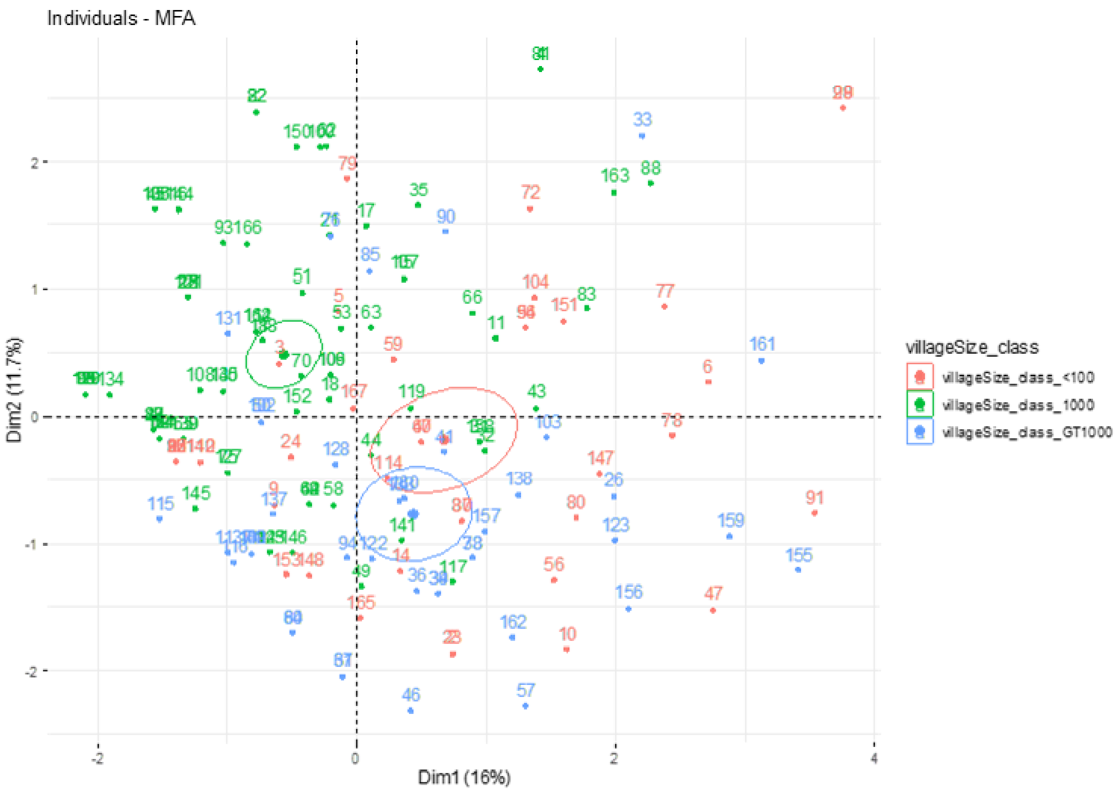

Figure S6. There is a weaker effect in this plot, with the middle village size (1000 = between 100 and 1000 inhabitants) generally having higher values for component 2 of the multifactorial analysis.

Figure S7

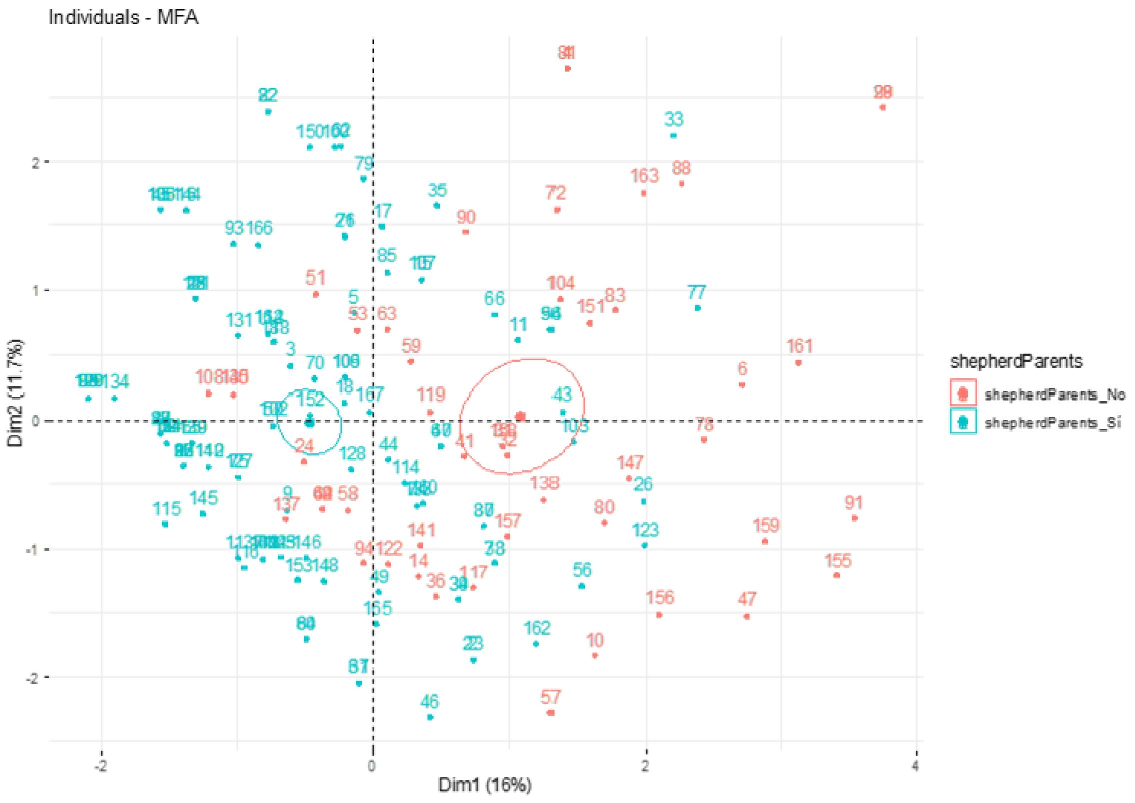

Figure S7. This plot represents whether herders had herder parents. The shows an association with component 1 (high values linked to not having herder parents) but this relationship is weak.

Figure S8

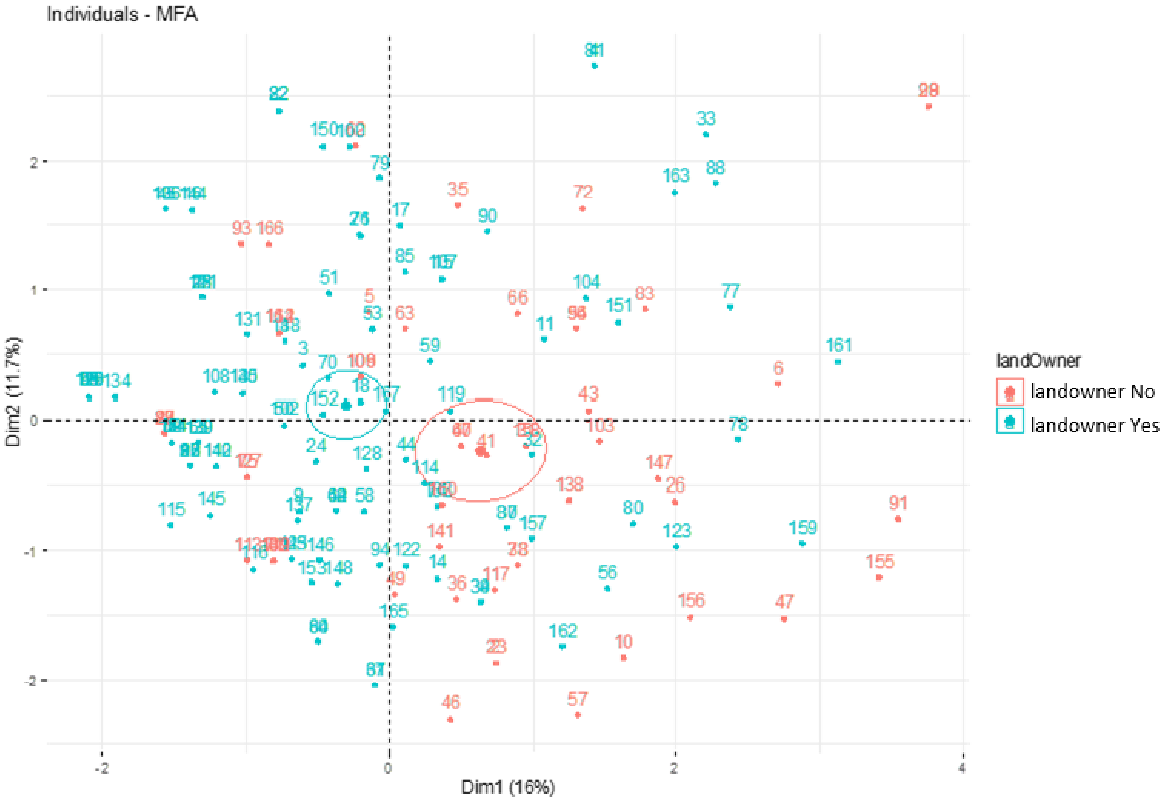

Figure S8. The plot shows a small association with component 1 (larger values linked with not owning land), but it is fairly weak.
